# Vessel Curvature and Microcatheter-Detachable Coil Compatibility in Arterial Coil Embolization

**DOI:** 10.7759/cureus.89699

**Published:** 2025-08-09

**Authors:** Kenichiro Okumura, Takahiro Ogi, Junichi Matsumoto, Nobuyuki Asato, Hirohito Osanai, Taichi Kitagawa, Atsushi Takamatsu, Fumihito Toshima, Kazuto Kozaka, Satoshi Kobayashi

**Affiliations:** 1 Department of Radiology, Kanazawa University Hospital, Kanazawa, JPN

**Keywords:** embolization, inerventional, microcatheter, microcoil, vascular

## Abstract

Background

Non-neuro arterial embolization is widely performed for various clinical indications, with support systems implemented to ensure procedural safety. However, when the target vessels are highly tortuous or have significant bends at the peripheral level, the microcatheter and coil must traverse lengthy, sharply curved paths, raising concerns about coil maldelivery.

Purpose

To examine whether sharp vessel angulation (≥90-180°) and microcatheter-coil compatibility independently increase coil maldelivery in non-neuroarterial embolization.

Materials and methods

This single-center, IRB-approved analysis from February 2023 to December 2024 included 451 arterial branch-detachable coil combinations (BCCs) in 119 patients (mean age 64 years, range 32-87). Angulations of 90-180° at the proximal catheter segment and distal tip were evaluated on digital subtraction angiography. Technical failure was defined as the inability to deploy the coil as intended, including unraveling or coil shape distortion. A 1:3 propensity score matching (28 failed vs 61 successful BCCs), balanced coil features, and target-vessel factors. Mismatch was recorded if the coil's primary diameter exceeded or fell below microcatheter thresholds. A generalized linear mixed model accounted for within-patient clustering.

Results

Coil failure occurred in 28 of 451 BCCs (6.2 %). A 90-180° inversion at the catheter tip (odds ratio (OR), 22; p = 0.008) and mismatch (OR, 4.9; p = 0.03) independently predicted failure. A proximal 90-180° inversion also contributed (OR, 4.5; p = 0.03). Of 28 failures, 21/28 (75%) were mismatched: 16/21 (76%) resolved via mismatch correction, and 5/21 (24%) required repositioning or an alternate coil gauge/length. Proper-match failures (n=7) were treated with thinner or more flexible coils in 6 (85.7%; p < 0.0001).

Conclusions

Sharp vessel angulation (≥90-180°) and microcatheter-coil mismatch independently increase the risk of maldelivery in non-neuro arterial embolization.

## Introduction

Non-neuro arterial embolization is widely performed to manage acute hemorrhage, tumor devascularization, and elective vessel occlusion in various vascular territories, including the gastrointestinal tract, genitourinary system, and musculoskeletal branches [1,2]. Among the many available embolic materials, coil embolization provides precise placement, retrievability, and controlled occlusion [3-5]. Nevertheless, unexpected coil maldelivery continues to occur, manifesting as coil deformation, unraveling, or misplacement, and is frequently linked to pronounced vessel curvature or a mismatch between the microcatheter’s inner diameter and the coil’s primary diameter (microcatheter-detachable coil mismatch) [6-11]. Moreover, the impact of specific anatomical factors, such as branch angulation and vessel tortuosity, on coil performance and complication risk remains only partially understood. Indeed, clinical observations indicate that even mismatches between microcatheters and coil primary diameter can impede smooth delivery, cause abnormal coil extension, or lead to unraveling, potentially compromising both technical success and patient outcomes [7]. Despite various coil and catheter designs, few large-scale investigations have systematically evaluated how anatomic curvature and device compatibility influence coil deployment in non-neuro branches.

Therefore, it was hypothesized that pronounced vessel angulation (≥90-180°) and a discrepancy between coil diameter and microcatheter lumen each heighten maldelivery. This retrospective study quantified these risk factors, emphasizing immediate technical outcomes and salvage methods.

## Materials and methods

Study design and oversight

This single-center retrospective analysis (February 2023-December 2024) was approved by our Institutional Review Board (approval number 2125-4). The requirement for written informed consent was waived in accordance with institutional guidelines. All procedures were conducted in accordance with institutional protocols for research ethics and the tenets of the Declaration of Helsinki. Consecutive patients requiring non-neuro-arterial embolization during the study period were identified.

Patient and procedure selection

**Figure 1:**
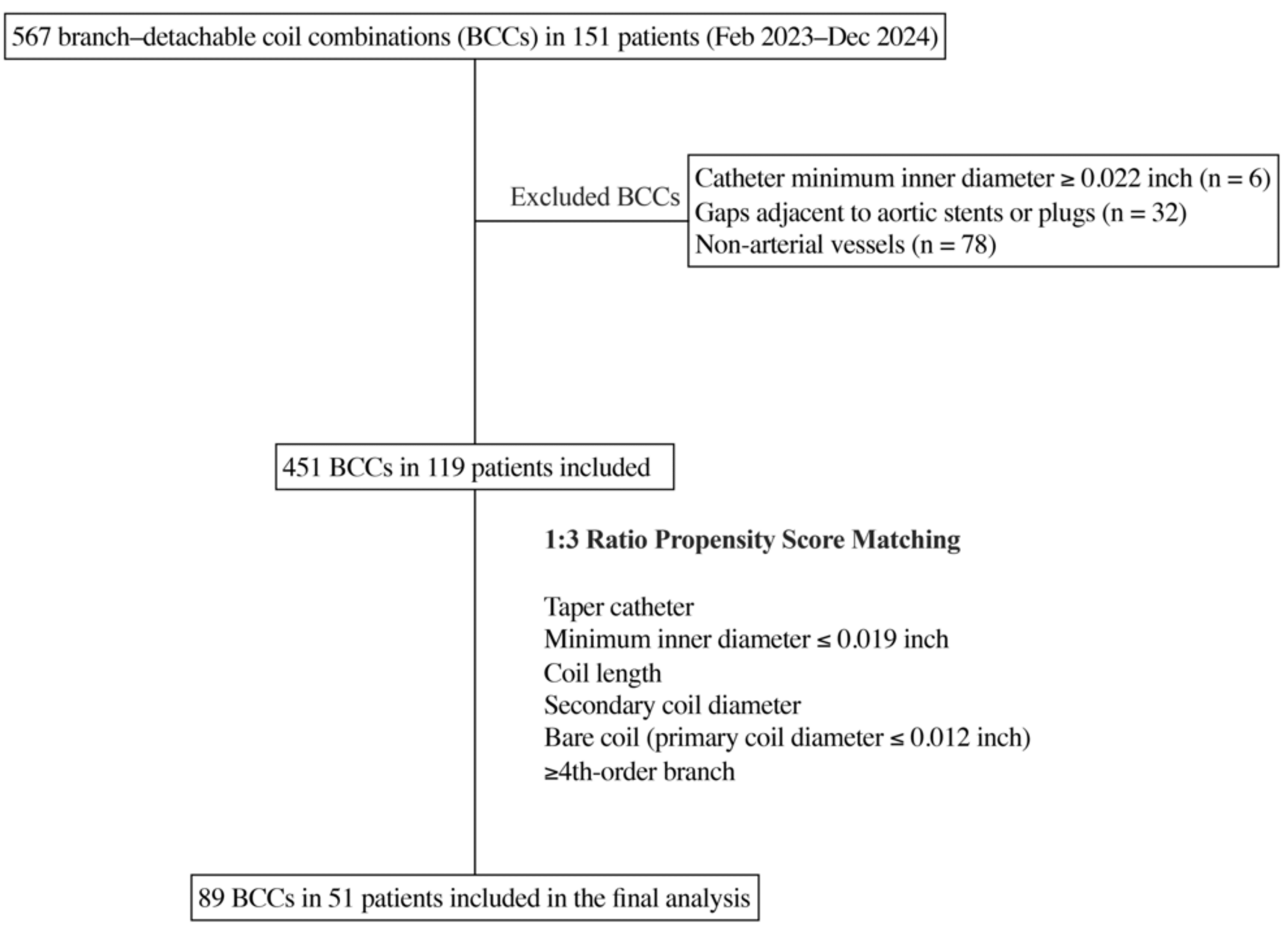
Flowchart of patient enrollment, exclusion, and propensity score matching

A total of 567 arterial branch-coil combinations (BCCs) were initially identified in 151 patients. Cases were excluded based on three criteria: (1) use of only high-flow catheters (≥0.022-inch inner diameter) (6 BCCs), (2) presence of stent or plug gaps around the aorta (32 BCCs), and (3) targeting of non-arterial vessels (venous or portal, 78 BCCs). After these exclusions, 451 BCCs remained in 119 patients (mean age, 64 years; range, 32-87). The sample size for this study was determined based on the availability of institutional data within the study period and feasibility considerations, rather than a predefined power analysis. While the number of cases provides a reasonable basis for statistical analysis, the relatively small number of failures (28 cases) may limit statistical power, and results should be interpreted accordingly. Figure 1 provides a detailed flowchart of patient enrollment, exclusion, and propensity score matching to illustrate the selection of the final study cohort. The procedures were performed by four attending interventional radiologists, each with over a decade of experience. The study was designed to focus on both procedural success and patterns of failure, particularly in relation to vascular morphology and coil-catheter compatibility.

Definition of technical failure

Technical success was achieved when the coil was fully deployed in the intended segment without requiring retrieval. Technical failure was documented if the coil (1) could not exit the catheter, (2) exhibited major unraveling or “stretching,” or (3) failed to assume a stable shape (e.g., extreme elongation) that required replacement. Although “failure to assume intended shape” can be subjective, procedural notes typically describe such events. This real-world approach inherently incorporates some observer bias but mirrors operator decision-making.

Microcatheter-detachable coil mismatch

Microcatheter-detachable coil mismatch was defined as any microcatheter-coil pairing that exceeded either the manufacturer’s indications or the additional criteria implemented in this study, which were derived from multiple product specifications. In practice, bare coils up to 0.012 inch generally fit microcatheters of 0.019 inch or less, whereas coils of 0.014 inch or larger require catheters of up to 0.021 inch. Fibered coils typically need a microcatheter diameter of at least 0.021 inch, and hydrogel polymer coils (AZUR Soft 3D, Terumo, Tokyo, Japan) are recommended for catheters with an inner diameter of approximately 0.0165-0.021 inch. Any deviation from these thresholds or from explicit manufacturer guidelines was classified as a mismatch. For example, using a 0.020-0.021-inch microcatheter with 0.010-0.012-inch bare coils may allow excess snaking, while placing a 0.012-0.015-inch fibered coil in a 0.019-inch microcatheter can increase frictional forces. In either case, suboptimal coil-catheter compatibility risks procedural difficulty or failure.

Microcatheter and detachable coil descriptions

A variety of taper and non-taper microcatheters were employed, chosen based on factors such as vessel size and operator preference. Taper models, including BISHOP (Piolax, Yokohama, Japan), Progreat λ19/λ17 (Terumo, Tokyo, Japan), and Velouté Ultra/19 DM (Asahi Intecc, Aichi, Japan), generally exhibited a minimum inner diameter between 0.015 and 0.019 inch. Non-taper models, such as MARVEL (Tokai Medical Products, Kasugai, Japan), Excelsior SL10 (Stryker, Fremont, CA, USA), and Lighthouse (Piolax, Yokohama, Japan) had inner diameters in the range of 0.0165 to 0.021 inch. Detachable coils covered a wide range of bare, fibered, and hydrogel polymer types, including Target Ultra, Tetra, and Nano coils (all 0.010 inch, Stryker, Fremont, CA, USA), as well as fibered coils like Interlock (0.012 inch) and Embold (0.015 inch) from Boston Scientific (Marlborough, MA, USA). Secondary coil diameter sizing is generally aimed at a diameter of approximately 10-20% larger than the vessel caliber to ensure adequate wall apposition. In most procedures, microcatheters and coils were selected according to institutional or manufacturer guidelines for compatibility (i.e., pairing microcatheter inner diameter and primary coil diameter within the recommended range). However, in certain emergent cases or when inventory constraints arose, coil-catheter combinations exceeding these recommendations were occasionally used. For the purposes of this study, such instances were classified as “mismatch” as previously defined. Although these off-label combinations were not routinely selected, they were sometimes the only feasible option in urgent situations. For this study, “flexible coils” were not defined by a single parameter but were generally considered to be those with a smaller primary diameter (e.g., ≤0.012 inch), a bare rather than fibered design, or an inherently pliable construction. In particular, the AZUR Soft 3D coil (Terumo, Tokyo, Japan) was deemed relatively flexible because its pusher-coil interface includes a joint of comparable suppleness to the coil itself, and it accommodates a wide range of recommended catheter inner diameters (0.0165-0.021 inch). This broad compatibility and reduced stiffness were key factors in labeling it as a flexible option in challenging anatomies.

Vascular anatomy assessment

**Figure 2:**
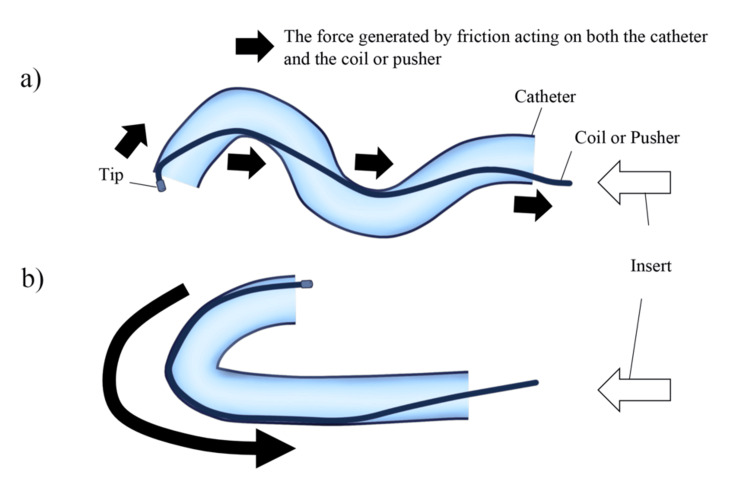
Schematic diagram of coil and coil pusher movement through a tortuous catheter, emphasizing frictional forces (a) The coil pusher moves through an S-shaped catheter, generating friction at contact points. (b) The coil pusher passes through a catheter with a 90–180° inversion, creating broad contact with the outer curve. This increased surface area leads to greater friction compared with the S-shaped configuration. Image credits: Dr. Kenichiro Okumura. (Data adapted from references [6-17])

**Figure 3:**
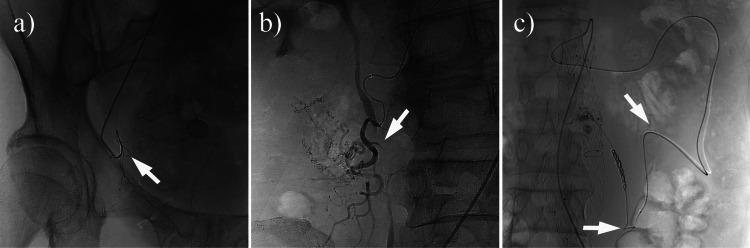
Representative fluoroscopic images showing the S-shaped and 90–180° inversion catheter configurations (a) Fluoroscopic spot image demonstrating a 180° inversion at the catheter tip (distal segment; arrow). (b) Fluoroscopic spot image revealing an S-shaped catheter configuration (arrow). (c) Fluoroscopic spot image illustrating a 180° bend in the proximal segment of the catheter (arrow).

Two interventional radiologists analyzed digital subtraction angiography (DSA) images to identify a proximal 90-180° inversion (Prox 90-180° Inv.) if the catheter formed a hairpin loop (≥90° but ≤180°) in the proximal path, or a tip 90-180° inversion (Tip 90-180° Inv.) if this occurred within the last 2 cm of the catheter tip. For this study, an “S-shaped configuration” was defined as two or more consecutive curves within the distal 2 cm of the catheter tip that together formed an approximate “S” shape in a single angiographic projection. The study also incorporated a quantitative evaluation of tortuosity, calculated as a meandering index (i.e., the ratio of the traced vessel path length to the straight-line distance between the proximal and distal landmarks) [12]. Higher values in this index indicate greater curvature. To clarify how frictional forces arise when the coil pusher travels through tortuous paths, a schematic diagram (Figure 2) illustrates the S-shaped and 90-180° inversion configurations. Representative fluoroscopic images further illustrate these configurations in actual procedures (Figure 3).

Management strategies for failed cases

Operators sometimes encountered difficulties in coil delivery but were able to salvage the procedure through various strategies. In many instances involving a clear mismatch, the solution involved exchanging the coil for another whose primary diameter or length better conformed to the microcatheter’s specifications. In other situations, the catheter was repositioned to avoid sharp angulations, and a more flexible coil was used to overcome a 90-180° inversion. The decision to select a more pliable coil design was commonly made after observing persistent frictional resistance or excessive snaking on angiography. By modifying the catheter tip position or coil attributes in real time, operators were frequently able to salvage the procedure and achieve stable coil formation within the target vessel.

Propensity score matching and statistical analysis

Because only 28 of 451 BCCs (6.2%) failed, a 1:3 nearest-neighbor propensity score matching (with replacement) was performed, balancing coil gauge (0.010-0.012 vs ≥0.014 inch), coil length, taper vs non-taper catheter, microcatheter inner diameter (≤0.019 vs >0.019 inch), and target branch level (≤3rd vs ≥4th order). In this study, branch levels were defined by sequentially counting each division from the aorta: arteries originating directly from the aorta were designated as first-order, their immediate offshoots as second-order, and so forth. Consequently, the celiac artery, common hepatic artery, splenic artery, left gastric artery, gastroduodenal artery, proper hepatic artery, renal artery, intercostal-lumbar arteries, superior and inferior mesenteric arteries, the inferior epigastric artery, the internal thoracic artery, the lingual and maxillary arteries, and the internal iliac artery were categorized up to third-order, and the right gastric artery fell under the fourth-order category based on the definition. Because actual embolization targets often lie distal to these major vessels, each target’s branch level was determined accordingly.

Subsequently, 28 failed and 61 successful BCCs were compared. Fisher’s exact test or t-tests/Mann-Whitney U tests were used for univariate comparisons. A generalized linear mixed model logistic regression (random intercept by patient) was used for multivariable analysis, with proximal 90-180° inversion, tip 90-180° inversion, S-shaped tip, quantitative tortuosity, and mismatch as covariates. Odds ratios (OR) and 95% confidence intervals (CI) were calculated, with p < 0.05 denoting significance. Analyses were conducted using GraphPad Prism (10.4.1) (GraphPad Software, Boston, MA, US), Python (3.13) (https://www.python.org/), and R (4.2.2) (R Core Team. R Foundation for Statistical Computing, Vienna, Austria).

## Results

Baseline characteristics

Before propensity score matching (PSM), 28 failed BCCs from 12 patients and 423 successful BCCs from 107 patients were identified. Table 1 presents the baseline characteristics and standardized mean differences (SMD) before and after 1:3 nearest-neighbor matching with replacement, showing that the SMD values in key variables (e.g., patient age, catheter type, coil length, and coil diameters) were substantially reduced in the matched dataset. As a result, the final analysis included 28 failed BCCs and 61 matched successful BCCs, indicating improved balance between the failure and success groups compared with the pre-matching cohort.

**Table 1:** Patient- and branch-coil-level characteristics before and after propensity score matching Characteristics are presented at the patient and branch-coil combination (BCC) levels. Patient numbers are listed alongside BCCs to clarify the scale of analysis. Standardized mean differences (SMDs) evaluate the balance between success and failure groups before and after propensity score matching.

Characteristics	Failure (n = 28 BCCs, 12 patients)	Pre-Matching Success (n = 423 BCCs, 107 patients)	SMD (Pre)	Post-Matching Success (n = 61 BCCs, 39 patients)	SMD (Post)
Patient level					
Age (years), mean (SD)	64 (14)	70 (13)	0.48	66 (15)	0.12
Male, n (%)	6 (50.0)	80 (74.8)	0.51	27 (69.2)	-0.08
Branch-coil level					
Total BCCs	28	423	-	61	-
Catheter; Taper, n (%)	7 (25.0)	37 (8.7)	-0.68	6 (10.0)	0.53
Catheter; Minimum inner diameter ≤ 0.019 inch, n (%)	5 (17.9)	36 (8.5)	0.04	22 (36.1)	0.02
Coil length (cm), mean (SD)	10 (4.4)	15 (11.6)	0.52	11 (5.4)	0.07
Secondary coil diameter (mm), mean (SD)	3.0 (0.9)	4.6 (2.9)	0.73	3.3 (1.1)	0.27
Primary coil diameter ≤ 0.012 inch, n (%)	9 (32.1)	27 (6.4)	0.94	32 (52.5)	0.45
Branches; ≥4th-order branch, n (%)	6 (21.4)	35 (8.3)	-0.78	36 (59.0)	0.18

Predictors of procedural failure

**Figure 4:**
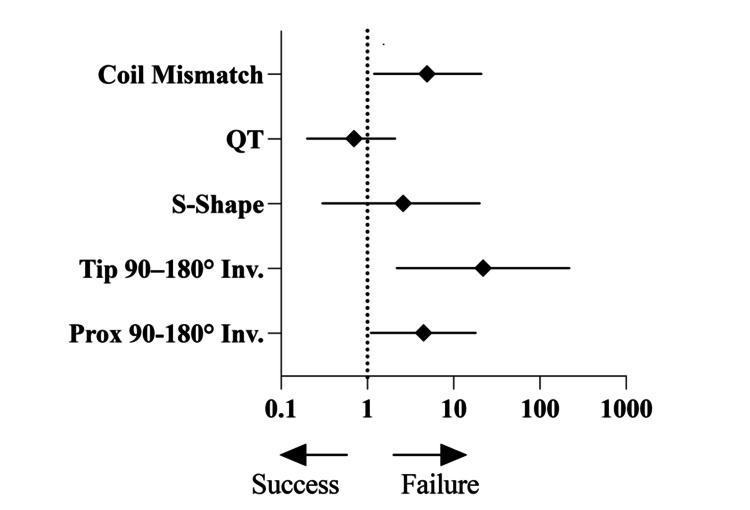
Forest plot of multivariate logistic regression results Forest plot showing adjusted odds ratios (◆) with 95% confidence intervals (horizontal lines) from the multivariate logistic regression model comparing procedural failure (n=28) to success (n=61). The vertical dashed line represents the reference line (OR=1). Variables left of the line (<1) favor success; variables right of the line (>1) favor failure. Abbreviations: Prox 90–180° Inv. = 90–180° inversion in the proximal portion of the catheter (excluding the tip); Tip 90–180° Inv. = 90–180° inversion of the distal 2 cm of the catheter tip; S-Shape = S-shaped configuration; QT = Quantitative Tortuosity; Coil Mismatch = Catheter inner diameter–coil primary diameter mismatch.

Table 2 and Figure 4 summarize the association between specific catheter configuration factors and procedural outcomes. In univariable analyses, proximal 90-180° inversion (Prox 90-180° Inv.), tip inversion from 90° to 180° (Tip 90-180° Inv.), the presence of an S-shaped configuration (S-Shape), and microcatheter-detachable coil mismatch were all significantly more frequent in failed cases (p-values of 0.01, <0.0001, <0.0001, and 0.01, respectively). Higher quantitative tortuosity (QT) was also noted in failed cases (mean ± SD, 1.8 ± 0.9 vs. 1.2 ± 0.5; p < 0.0001). However, in the generalized linear mixed model (GLMM)-based logistic regression, only Prox 90-180° Inv. (p = 0.03; OR, 4.5; 95% CI, 1.1-18), Tip 90-180° Inv. (p = 0.008; OR, 22; 95% CI, 2.2-220), and microcatheter-detachable coil mismatch (p = 0.03; OR, 4.9; 95% CI, 1.2-21) remained independently associated with higher odds of procedural failure. Neither the S-shaped configuration nor quantitative tortuosity showed a significant independent effect after adjusting for other factors.

**Table 2:** Logistic regression results for catheter configuration and tortuosity variables Prox 90–180° Inv. = 90–180° inversion in the proximal portion of the catheter (excluding the tip); Tip 90–180° Inv. = 90–180° inversion of the distal 2 cm of the catheter tip; S-Shape = S-shaped configuration; Coil mismatch = Catheter inner diameter–coil primary diameter mismatch; QT = Quantitative Tortuosity. Univariate p-values were obtained by Fisher’s exact test (categorical variables)/Mann–Whitney U test (continuous variables). Multivariate p-values, odds ratios (OR), and 95% confidence intervals (CI) were derived from the generalized linear mixed model (GLMM) logistic regression. Test statistics are reported as U-values for Mann–Whitney U tests; Fisher’s exact test did not yield a test statistic and is therefore marked as “–”.

	Failure n (%) N = 28	Success n (%) N = 61	Univariable test statistic	Univariable p-value	Multivariable p-value	OR (95% CI)
Intercept	-	-		-	0.001	0.04 (0.005-0.25)
Prox 90-180° Inv.	17 (60.7%)	19 (31.1%)	-	0.01	0.03	4.5 (1.1-18)
Tip 90–180° Inv.	21 (75.0%)	6 (10.0%)	-	<0.0001	0.008	22 (2.2-220)
S-Shape	15 (53.6%)	3 (5.0%)	-	<0.0001	0.36	2.6 (0.3-20)
QT, mean (SD)	1.8 (0.9)	1.2 (0.5)	U = 288.5	<0.0001	0.55	0.7 (0.2-2.1)
Coil mismatch	21 (75.0%)	27 (44.3%)	-	0.01	0.03	4.9 (1.2-21)

Management strategies for failed cases

Table 3 summarizes the management approaches for the 28 failed branch-coil combinations, including resolution of microcatheter-detachable coil mismatch (RCM), repositioning the catheter or choosing an alternate coil gauge/length, and using thinner or more flexible coils. Among the 28 failed procedures, 21 (75%) were categorized as microcatheter-detachable coil mismatch and 7 (25%) as proper match. Three main management strategies were employed to address these failures: (1) resolution of the mismatch (RCM), (2) repositioning the catheter tip or selecting an alternate coil gauge/length, and (3) using a thinner or more flexible coil. Of the 21 mismatch failures, 16 (76%) were salvaged by RCM, whereas 5 (24%) required catheter-tip repositioning or a different coil dimension. In contrast, 6 of the 7 proper-match failures (85.7%) were managed by switching to thinner or more flexible coils. This distribution differed significantly between the mismatch and proper-match groups (p < 0.0001).

**Table 3:** Management strategies for 28 failed branch-detachable coil combinations RCM = Resolution of microcatheter–detachable coil mismatch; Catheter Repositioning or Alternate Coil = Microcatheter repositioning to avoid kinking or selection of a different coil gauge/length; Thinner or More Flexible Coil = Use of a coil with thinner strands or greater flexibility

Mismatch Category	RCM (n = 16), n (%)	Catheter Repositioning or Alternate Coil (n = 6), n (%)	Thinner or More Flexible Coil (n = 6), n (%)	P-value
Microcatheter-detachable coil mismatch (n =21)	16/16 (100%)	5/6 (83.3%)	0/6 (0%)	<0.0001
Proper microcatheter-detachable coil match (n=7)	0/16 (0%)	1/6 (16.7%)	6/6 (100%)	

## Discussion

When a microcatheter navigates a sharply angled vascular path (90-180° inversions), friction between the coil and the catheter wall increases, impeding smooth insertion and potentially exceeding the coil’s column strength. Prior in vitro studies have demonstrated that severe angulations generate substantial frictional forces, predisposing weaker coil segments to deform [6,7,9,10,13]. In line with those findings, the present study revealed that sharply angulated anatomy alone, not just microcatheter-detachable coil mismatch, elevates the risk of maldelivery, underscoring the influential role of geometric factors. From a biomechanical standpoint, sharp vascular angulation inherently magnifies frictional stress between the microcatheter and coil. When the bend is acute (e.g., ≥90°), the coil is forced to conform tightly against the catheter wall, particularly when the delivery system’s support is compromised due to the tortuous access path. In such cases, even if the microcatheter itself is appropriately selected, the transmission of axial force to the coil tip may be insufficient, leading to backward movement or “kickback.” This suggests that failures may not only be due to the vascular angle itself, but also to the mismatch between the coil stiffness and the effective mechanical support of the entire delivery system. Prior in vitro studies, albeit focused on neurovascular models, have demonstrated that steeper angles and narrow lumens can amplify axial loading on the coil, causing it to stretch, unravel, or fail to form its intended configuration. Put differently, if either the vessel angle is too sharp or the coil is too large relative to the microcatheter diameter, frictional forces are likely to escalate, jeopardizing smooth delivery.

A mismatch between the microcatheter lumen size and the detachable coil primary diameter can amplify frictional challenges. Previous studies have indicated that a narrow lumen creates excessive resistance, potentially causing the coil to deform or unravel, whereas an oversized lumen may lead to uncontrolled movement and reduced coil elasticity, thereby increasing the likelihood of snaking [9,10,14]. Forced coil insertion under either scenario can cause the catheter to retract or “kick back,” preventing proper coil placement. The present analysis similarly found that mismatch was a significant risk factor, present in 75% of all failures. Among these mismatch-related failures (n=21), 16 (76%) were salvaged by resolving the mismatch (RCM), while 5 (24%) required catheter-tip repositioning or a different coil gauge/length (p < 0.0001). This outcome suggests that even when an initial mismatch exists, reducing the vascular sharpness (e.g., repositioning the catheter to avoid acute bends) or adjusting coil length/diameter can help the coil deploy more linearly and prevent further complications. However, even properly matched systems faced difficulties when encountering acute angulations, suggesting that geometric factors can outweigh device compatibility alone.

Prior bench experiments have reported that a tight microcatheter-coil fit in an acutely curved model leads to excessive friction and coil distortion [9,10,14-17]. In these bench simulations, coils navigating a steep angle frequently showed partial unraveling or shape compromise, mirroring our clinical observations in branches with ≥90° angulation. Thus, existing in vitro data provide a plausible mechanistic explanation for our findings, even though our investigation focused on real-world procedures rather than controlled laboratory conditions. By referencing these previous bench models, we underscore that the interplay of sharp angles and marginal coil-catheter clearance is not merely correlational but stems from heightened mechanical stress.

Several limitations merit consideration. First, the definition of coil failure was partly subjective. Second, vessel angulation was determined using two-dimensional DSA rather than three-dimensional reconstructions, potentially underestimating the true severity of curvature. Third, high-flow catheters were excluded, limiting the generalizability of these findings to those devices. Fourth, the small number of failures (n=28) constrained statistical power. Fifth, although PSM reduced many baseline imbalances, some SMDs remained above 0.2, suggesting possible residual confounding. Nonetheless, the SMDs were substantially lower than before matching, indicating partial improvement in cohort comparability. Finally, many initial failures were ultimately salvaged by repositioning the catheter or switching to a more flexible coil, indicating that maldelivery does not necessarily preclude successful embolization. 

## Conclusions

In conclusion, this single-center retrospective study investigated 451 arterial branch-coil combinations in non-neuro embolization and identified two independent predictors of coil maldelivery: sharp catheter angulations (≥90-180°) and microcatheter-coil mismatch. In particular, tip inversions with acute angulation were associated with significantly increased failure risk (OR=22), underscoring the importance of preprocedural anatomical evaluation. Recognition of these anatomical and technical risk factors can improve procedural planning and reduce complications by informing appropriate device selection. Moreover, approximately 75% of failures were due to a mismatch, and most of these were successfully salvaged by either adjusting coil dimensions or repositioning the catheter. These findings emphasize the need for intra-procedural flexibility and may inform the development of future embolization protocols, especially in tortuous vascular territories.

While limited by its retrospective nature and small number of failure events, this study provides actionable insights for interventionalists managing technically complex embolization cases. A better understanding of device-vessel interactions may help minimize maldelivery and improve success rates in challenging vascular anatomy. Even in cases without apparent mismatch, coil maldelivery occurred in the presence of acute tip angulation. In such instances, the use of thinner or more flexible coils proved effective in most cases. These findings suggest that geometric complexity alone may necessitate preemptive adjustments in device strategy, including catheter tip repositioning or the selection of coils with reduced stiffness.
